# Design of Improved Flow-Focusing Microchannel with Constricted Continuous Phase Inlet and Study of Fluid Flow Characteristics

**DOI:** 10.3390/mi13101776

**Published:** 2022-10-19

**Authors:** Zhaohui Wang, Weibing Ding, Yiwei Fan, Jian Wang, Jie Chen, Hongxia Wang

**Affiliations:** 1 Key Laboratory of Metallurgical Equipment and Control Technology of Ministry of Education, Wuhan University of Science and Technology, Wuhan 430081, China; 2 The State Key Laboratory of Fluid Power and Mechatronic Systems, Zhejiang University, Hangzhou 310027, China; 3 College of Mechanical Engineering, Hubei University of Automotive Technology, Shiyan 442002, China

**Keywords:** flow-focusing microchannel, RBF model, microbubbles, optimal design, flow characteristics

## Abstract

This paper proposed an improved flow-focusing microchannel with a constricted continuous phase inlet to increase microbubble generation frequency and reduce microbubbles’ diameter. The design variables were obtained by Latin hypercube sampling, and the radial basis function (RBF) surrogate model was used to establish the relationship between the objective function (microbubble diameter and generation frequency) and the design variables. Moreover, the optimized design of the nondominated sorting genetic algorithm II (NSGA-II) algorithm was carried out. Finally, the optimization results were verified by numerical simulations and compared with those of traditional microchannels. The results showed that dripping and squeezing regimes existed in the two microchannels. The constricted continuous phase inlet enhanced the flow-focusing effect of the improved microchannel. The diameter of microbubbles obtained from the improved microchannel was reduced from 2.8141 to 1.6949 μm, and the generation frequency was increased from 64.077 to 175.438 kHz at the same capillary numbers (Ca) compared with the traditional microchannel. According to the fitted linear function, it is known that the slope of decreasing microbubble diameter with increasing Ca number and the slope of increasing generation frequency with increasing Ca number are greater in the improved microchannel compared with those in the traditional microchannel.

## 1. Introduction

Nowadays, microbubbles have become one of the most effective contrast agents in medical ultrasound imaging or as a carrier for targeted drug delivery [1,2,3,4,5,6,7]. Generally, the required microbubble diameter must be between 1 and 7 μm [8]. Currently, there are many ways to prepare microbubbles. Due to its high throughput and low cost, ultrasound is commonly used to generate microbubbles. Still, the ultrasound generator’s frequency, power, and pulse affect the size of the microbubbles and cannot produce uniform microbubbles of a specific size, which requires an additional filtering process to remove larger bubbles [9]. The mechanical stirring method [10] prepares microbubbles by mechanical stirrers but produces microbubbles of uneven size. The inkjet printing method [11] allows better control of the size of microbubbles but is limited to the production of liquid-filled particles. The coaxial electrofluidic atomization method [12] produces microbubbles with diameters less than 10 μm, but it also produces microbubbles of uneven size. All of the above methods produce polydisperse microbubbles in liquids, limiting the potential use of microbubbles in medicine. In contrast, microfluidics can produce highly monodisperse microbubbles [13,14].

Microfluidics has been used as a promising interdisciplinary technique for generating and manipulating monodisperse and discrete micron-sized bubbles. It is possible to reduce the size of the generated microbubbles by adjusting the flow parameters and narrowing the orifice structure to produce microbubbles with very narrow dimensions. It has the advantages of monodisperse bubble generation, easy control of bubble size, small sample volume, and low cost. Standard microfluidic techniques for generating microbubbles can be divided into three main types: the T-type, coaxial flow type, and flow-focusing type. The T-joint device has a simple structure, including liquid and gas channels that vertically intersect with the liquid channel [15]. Microbubble formation in the T-joint can be divided into three regimes: squeezing, dripping, and transiting [16]. The generation of microbubbles less than 10 μm in diameter for typical T-type structures is still problematic [17]. Coaxial flow devices usually have an exit orifice wider than the microbubble diameter. The liquid velocity is always high enough, so liquid inertia is the dominant force for microbubble formation. The coaxial flow device is characterized by a thin capillary tube supplying the gas surrounded by a coaxially arranged capillary tube supplying the liquid [18]. Flow-focusing microchannels usually consist of a central gas channel, two lateral liquid channels, and a narrow exit hole [19]. Flow-focusing microchannels use a constriction hole downstream of the dispersed phase’s inlet to modify the flow field’s hydrodynamic behavior and, thus, the formation of microbubbles. Compared with other microchannels, the microbubble generation in flow-focusing microchannels is easier to control. The bubble generation is relatively stable, efficient, and well-homogenized and has a more extensive adjustable size range. Therefore, it has attracted a lot of attention from researchers. Some experimental and numerical studies have been conducted.

Numerous visualization experiments have been conducted to investigate how microbubbles are produced in microchannels. Dietrich et al. [20] used flow-focusing microdevices to generate bubbles and measure the flow field with the micro-PIV technique. They found that the formation of bubbles depends on the geometry of the intersection and that the shear force was higher at 180° than at 60°, which indicates that the shear stress is more pronounced when the angle increases. In addition, the bubbles were more elongated in the 60° geometry than in the 180° geometry, suggesting an increase in surface tension due to deformation as the angle of connection decreases. Fu et al. [21] experimentally observed that tiny double bubbles could be formed in flow-focusing microchannels from a highly deformed gas conical jet at high liquid flow rates, whereas the gaseous jet in the downstream channel was stable. Castro-Hernández et al. [22] proposed a new mode of operation for the flow-focusing microfluidic devices, producing monodisperse bubbles with a diameter of 5 µm at a production rate of more than 10^5^ Hz by properly adjusting the flow rates of the gas and liquid. Sheng et al. [23] first reported the generation of short bubbles at high frequency in capillary embedded step T-junction microdevices, and the mechanism of short bubble formation is mainly because of the relatively high pressure drop inside the step T-junction, which provides a much higher breakup force for the squeezing flow. The above experiments provide a visual view of microbubble generation and limited insight into the hydrodynamic mechanism of microbubble generation.

In addition to experimental methods, numerical simulations based on computational fluid dynamics methods provide detailed fluid dynamics information and become an effective tool for studying multiphase fluid flows. Jia and Zhang [24] conducted a three-dimensional numerical study of the bubble formation process in viscous fluids and found that the geo-reconstruction method performs better. Zadeh and Radespiel et al. [25] investigated the effects of two-phase flow rate, two-phase viscosity, surface tension, and geometrical channel characteristics (channel width and injection angle) on microbubble formation and its length based on the volume of fluid (VOF) method. The results showed that at a constant flow rate in the continuous phase, the bubble length significantly increased with an increasing flow rate in the dispersed phase; the bubble length increased as the injection angle increased from 45° to 90°. Chekifi et al. [26] used the coupled level set and volume of fluid (CLSVOF) method to track the interface and showed that the velocity ratio, interfacial tension, outer channel diameter, continuous phase viscosity, orifice width, and length have important effects on the bubble size and shape. At lower capillary numbers, an increase in the flow velocity ratio produces smaller bubble sizes in a shorter time, whereas an increase in the interfacial tension produces larger bubbles.

In summary, most researchers have studied the two-phase flow from its operational parameters and physical properties. Most early studies only considered the role of individual geometrical parameters without integrating the effect of geometrical parameters on microbubble generation. There was a lack of studies on the optimal design of flow-focusing microchannels. Geometry in flow-focusing devices plays an essential role in microbubbles’ size and generation frequency. In addition, these effects are necessary for the optimal design of microbubble generation devices. Therefore, it is crucial to study the geometry of flow-focusing microchannels.

This paper proposed an improved flow-focusing microchannel with constricted continuous phase entrance. A two-dimensional numerical model of microbubble generation in a flow-focusing microchannel was established using the VOF method. Four design variables of the microchannel were selected, and the radial basis function (RBF) surrogate model was used to establish the relationship between the objective function (microbubble diameter and generation frequency) and design variables. Based on this, the NSGA-II algorithm’s optimal design was carried out. Finally, the optimization results were verified by numerical simulations, and smaller microbubbles with higher generation frequency were obtained in comparison with the traditional microchannels. The fluid flow characteristics of microchannels were also further studied to investigate the effect of microchannels on microbubble generation.

## 2. Model Description

### 2.1. Structural Design of Flow-Focusing Microchannels

**Figure 1:**
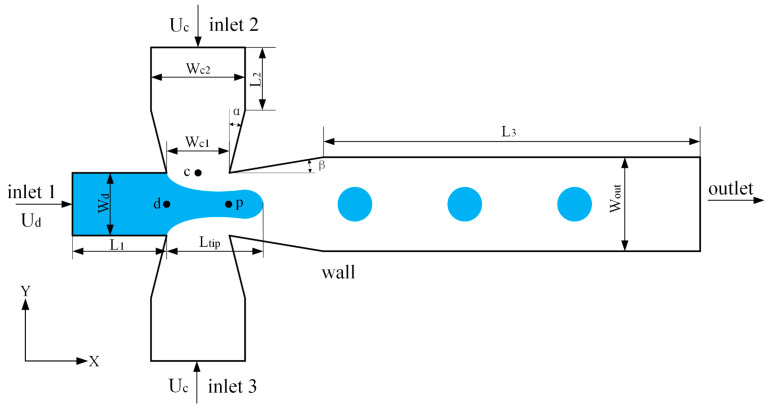
Schematics for improved flow-focusing microchannel.

The flow-focusing microchannel is a typical axisymmetric microchannel, consisting of a dispersed phase inlet and two continuous phase inlets to form focusing holes. The microbubble generation is achieved by passing the dispersed and continuous phases through the focusing holes. The geometry of the flow-focusing microchannel is shown in Figure 1. The geometry is fully defined by six parameters, namely the orifice width ($W_{d}$), continuous phase inlet constriction width ($W_{c1}$), continuous phase inlet width ($W_{c2}$), outlet width ($W_{out}$), constriction angle (α), and expansion angle (β), where the orifice width is 2 μm and the continuous phase inlet width is 2$W_{d}$ = 4 μm. To facilitate the uniform adjustment of the structural parameters, the dispersed phase inlet width is kept the same as the orifice width, and the continuous phase inlet constriction width and outlet width are normalized to the orifice width as shown in Equations (1) and (2).

(1) $${\bar{W}}_{c1}=\frac{W_{c1}}{W_{d}}$$

(2) $${\bar{W}}_{out}=\frac{W_{out}}{W_{d}}$$

where ${\bar{W}}_{c1}$ is the normalized continuous phase inlet constriction width, and ${\bar{W}}_{out}$ is the normalized outlet width. The gas phase inlet is $L_{1}$=$2W_{d}$ upstream from the orifice, and the liquid phase inlet is $L_{2}$=$2W_{c2}$ from the constriction so that the flow is fully developed before entering the orifice. The outlet channel is located at $L_{3}$*=*$15W_{d}$ from the end of the exit expansion angle to ensure that the two-phase flow is fully developed in the downstream microchannel. The continuous phase inlet constriction width, outlet width, constriction angle, and expansion angle are selected as design variables, and the design variables and design space are shown in Table 1.

In this paper, water was selected as the continuous phase (its density and viscosity coefficient is $\rho_{c}$ = 998.2 kg/m^3^,$\;\mu_{c}$ = 1.003 mPa·s), nitrogen is the dispersed phase (its density and viscosity coefficient is $\rho_{d}$ = 1.138 kg/m^3^,$\;\mu_{d}$ = 0.01663 mPa·s), and the surface tension coefficient between the two phases is σ = 0.072 N/m. The Ca number (Ca=μυ/σ) is defined for the aqueous phase (continuous phase), where all physical properties of both phases remain constant, so the Ca number varies only with the flow rate. The straight passageway transports the gas phase (N_2_), and the liquid phase flow (water) penetrates into the main passageway in the connection area. At the junction, the gas phase transforms into bubbles. At the end of the fragmentation process, the generated bubbles flow in the straight channel to the outlet.

### 2.2. Numerical Model

The Reynolds number Re is less than 10 for all cases in the flow-focusing system [27]. Therefore, the flow can be considered a laminar flow. In the simulations in this paper, the VOF method was used to track the interface between the continuous and dispersed phases. Since the density and viscosity of the two-phase fluid are constant, it can be regarded as an incompressible isotropic Newtonian fluid, so the Navier–Stokes equations describing the conservation of its momentum control equations are shown below.

(3) ∇·ν=0

(4) $$\frac{\partial \nu }{\partial t}+\left(\nu \cdot \nabla \right)\nu =-\frac{1}{\rho }\nabla P+\frac{\mu }{\rho }\nabla \left(\nabla \nu +\nabla \nu^{T}\right)+\frac{1}{\rho }F$$

where ν is the velocity vector of the fluid; t is the time; P is the pressure; ρ and μ are the density and viscosity of the fluid; (ν·∇)ν is the inertial force per unit volume of fluid; ∇P is the pressure gradient per unit volume of fluid; $\mu \nabla \left(\nabla \nu +\nabla \nu^{T}\right)$ denotes the viscous force per unit volume of fluid; and F is the momentum source term associated with the surface tension.

The capture of the two-phase interface motion can be characterized by calculating the distribution of the continuous and dispersed phase volume fractions $\varphi_{c}$ and $\varphi_{d}$ in a grid cell, where $\varphi_{c}$ = 1 and $\varphi_{d}$ = 0 for the continuous phase and $\varphi_{d}$ = 1 and $\varphi_{c}$ = 0 for the dispersed phase. Therefore, the two intersecting interfaces in a calculation cell depend on the magnitude of $\varphi_{c}$ and $\varphi_{d}$, taking values between 0 and 1. In the two-phase mixing unit, the calculation of the two-phase mixing density and viscosity in Equations (3) and (4) can be obtained from Equations (5) and (6).

(5) $$\rho =\varphi_{d}\rho_{d}+\left(1-\varphi_{d}\right)\rho_{c}$$

(6) $$\mu =\varphi_{d}\mu_{d}+\left(1-\varphi_{d}\right)\mu_{c}$$

In addition, the volume fraction $\varphi_{d}$ can be obtained by solving the continuous equation for the volume fraction:

(7) $$\frac{\partial \varphi_{d}}{\partial t}+\nu \nabla \varphi_{d}=0$$

In the calculation grid adjacent to the wall surface, the two phases of liquid and gas are in contact with the wall surface at a fixed angle, and a contact angle model can be used to characterize the effect of wall wettability. The contact angle θ can be defined as the angle between the liquid–gas and solid–liquid phase interfaces at the intersection of the solid wall surface, liquid phase, and gas phase. The normal unit vector in the calculation cell close to the wall surface in this model can be obtained by processing Equation (8):

(8) $$\hat{n}={\hat{n}}_{w}\cos\theta +{\hat{t}}_{w}\sin\theta$$

where ${\hat{n}}_{w}$ and ${\hat{t}}_{w}$ represent the wall unit normal vector and tangential vector, respectively.

In addition to the control equations, the relevant boundary conditions of the flow-focusing microchannel are as follows:

1. Inlet: Both phases are uniform inlet, and the flow velocity of the continuous and dispersed phase inlets are set to 1 m/s and 0.2 m/s, respectively, to ensure that microbubbles can be generated in the dripping regime.
2. Outlet: The ambient pressure is used as the outlet fluid’s reference pressure, equal to 0 Pa.
3. Wall: The contact angle is set to 50°, and the channel wall is set to no-slip boundary condition.

In this study, a double-precision pressure solver was used for numerical simulations. The pressure implicit algorithm with operator splitting (PISO) is used to implement the pressure level coupling, which is based on a higher order of the approximate relationship between pressure and velocity corrections. Pressure staggering option (PRESTO) is chosen as the pressure interpolation algorithm; the momentum term is in the second-order windward form, and the volume fraction is in the geo-reconstruct format. Due to the high accuracy of curvature calculation, the geometric reconstruction format is used for interface interpolation. The convergence criterion is set to 1 × 10^−6^. In the simulation, the variable step size is automatically adjusted according to the criterion that the global Courant number is less than 0.25, thus maintaining the stability and convergence of the solution.

### 2.3. Grid Independence and Model Validation

**Figure 2:**
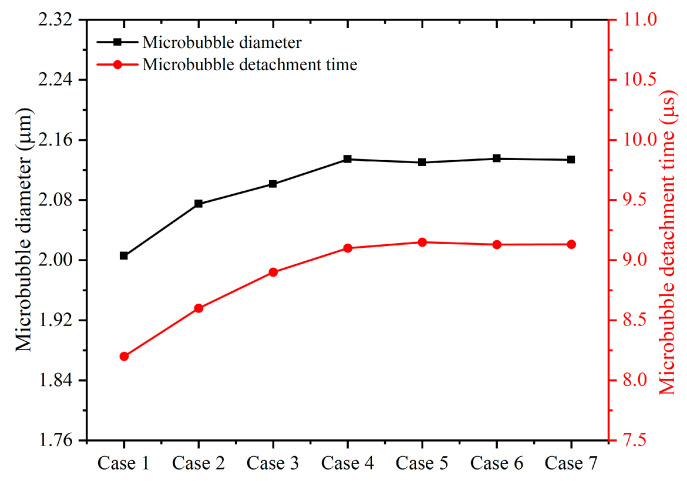
Grid independence test.

Usually, the smaller the grid is, the more accurate the calculation results are; however, the smaller the grid is, the more computational resources are consumed. In order to find the most economical mesh size, the unstructured mesh is used to mesh the 2D geometric model of the flow-focusing microchannel. Since different design variables affect the number of meshes, seven scale sizes ($\Delta h=W_{d}/10,W_{d}/15,W_{d}/20,W_{d}/25,W_{d}/30,W_{d}/35,W_{d}/40$*)* are selected for the meshing from cases 1 to 7. As shown in Figure 2, the variations between the minimum and maximum values of the microbubble diameter and microbubble detachment time are only 0.24% and 0.55%, respectively, from cases 4 to 7, indicating that the grid cells meet sufficient accuracy requirements. Therefore, to balance the accuracy of the calculation results and computational cost, Δh*=*$W_{d}/25$ grid cells are chosen for meshing the geometric channels in the subsequent simulations.

**Figure 3:**
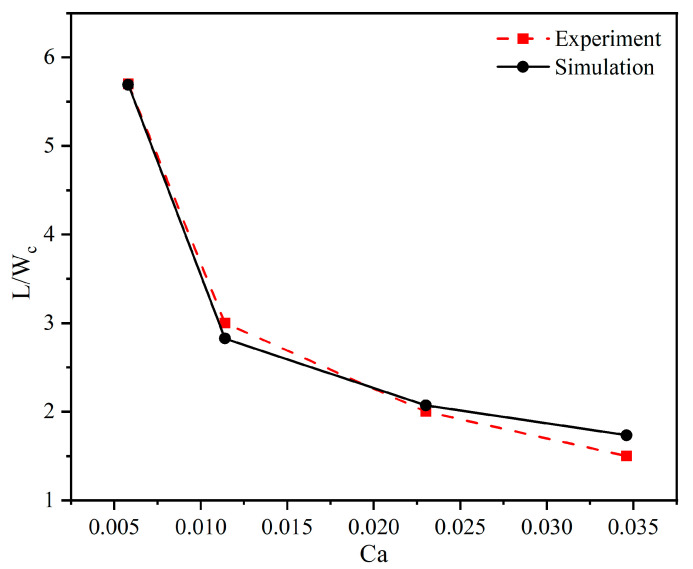
Comparison of numerical results of dimensionless microbubble size with experimental results.

In addition, to verify the accuracy of the current numerical simulation calculation method, the numerical simulation results were compared with the experimental results in [28], as shown in Figure 3. The figure shows that the microbubble formation results obtained from the numerical simulation agree well with the experimental results. The average error between the simulated and experimental values is 6.27%, which is within the acceptable range. Therefore, it can be concluded that the numerical simulation method used in this paper is accurate and feasible for characterizing the two-phase flow patterns within the flow-focusing microchannels.

## 3. Research Methods

### 3.1. Design of Sampling Point

The Latin hypercube sampling method was proposed in 1979 by M.D. Mc Kay, R.J. Beckman, and others as a constrained and homogeneous sampling method [29]. Its purpose is to ensure that the sampling points cover all sampling areas. Suppose that the sampling size is n; the number of variables is m; $x_{i}$ is the variable, and each variable is divided into n intervals of equal probability,$\;x_{i,l}=x_{i,0}<x_{i,1}<\ldots <x_{i,n}=x_{i,u}$; and $x_{i,l}$ and $x_{i,u}$ are the upper and lower bounds of the variable $x_{i}$, respectively. Extract a random sample from each interval of the variable $x_{i}$, combining the n points extracted from $x_{1}$ with the n points in $x_{2}$ in a random and nonrepetitive manner until finally combining them with the n sample points in the variable $x_{m}$. The sample points are designed according to the minimum distance maximum criterion, and the minimum distance d should satisfy

(9) $$\min\left(d\right)=max\left(min\left(d_{ij}\right)\right)$$

where $d_{ij}$ is the distance between sampling points $x_{i}$ and $x_{j}$:

(10) $$d_{ij}=\sqrt{\overset{m}{\underset{\mu =1}{\sum }}{\left|x_{i}{}^{\mu }-x_{j}{}^{\mu }\right|}^{2}}$$

The study used a total of four design variable parameters. Latin hypercube sampling was used to choose the 40 sampling sites that would provide a large enough sample of data. After the initial simulation test, a sampling point matrix of 4 × 40 was obtained, with each row representing a set of test parameters and each column representing a variable. The sample of the collected data obtained are shown in Table A1 in Appendix A.

### 3.2. Surrogate Model

A radial basis function is a neural network that includes an input layer of all influences, a hidden layer, and an output layer consisting of all responses [30,31,32]. The transfer function between the first two layers is a radial basis function, whereas the transfer function between the hidden and output layers is linear. RBF neural networks are characterized by fast training speed and compact networks.

**Figure 4:**
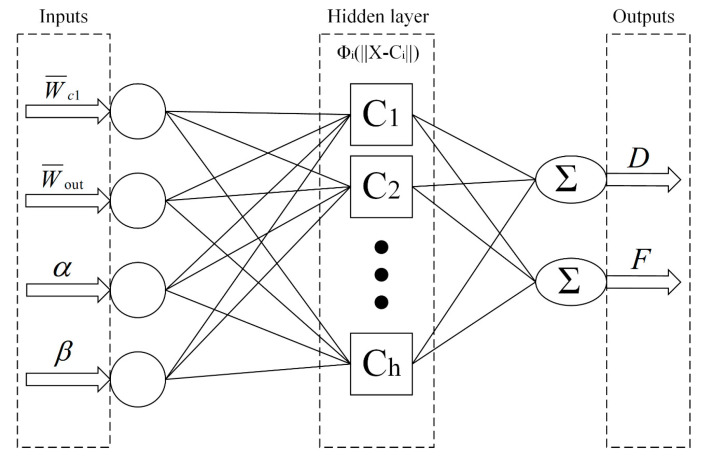
RBF neural network topology.

A surrogate model with design variable parameters (${\bar{W}}_{c1},{\bar{W}}_{out},\alpha ,\beta$) and the objective function after the response of each sample point was developed, where the optimization metrics included the diameter (D) and frequency (F). In this work, the four parameters of the design variables were used as variables in the input layer, and the response of the output layer was obtained by transforming the hidden layer. The RBF topology of this work is shown in Figure 4.

### 3.3. NSGA-II

When multiple objectives exist, especially competing for objective functions, single-objective optimization methods cannot meet the requirements. Therefore, there is a need for a multi-objective optimization method that can simultaneously optimize multiple objective functions. Genetic algorithms have good applications in solving multi-objective problems.

Kalyanmoy Deb et al. proposed an efficient and fast global optimization algorithm in 2001 inspired by the Darwinian evolutionary theory [33]. The nondominated sorting genetic algorithm II (NSGA-II) randomly generates the initial population based on the objective function and then performs nondominated sorting and crowding distance calculation. The new generation is obtained through crossover, selection, and mutation by genetic algorithms. These progeny populations are combined with the initial parent population for nondominated sorting and calculation of the crowding distance for each individual. The best individuals are selected based on sorting and crowding distance to form the initial population of the next generation. This is a cyclic process until a certain number of evolutionary generations are reached, or the convergence criterion is satisfied, and the whole optimization process ends.

**Figure 5:**
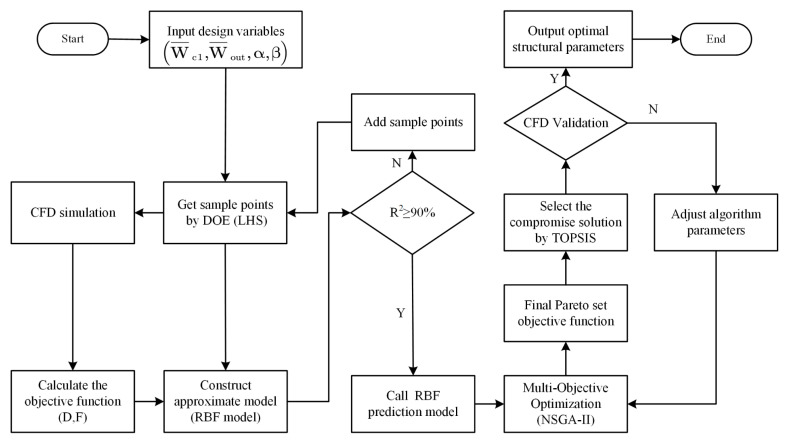
Multi-objective optimization flow chart.

NSGA-II has good robustness and convergence. It ensures a uniform distribution of non-inferior optimal solutions, which are less likely to fall into local optima during the search process. It is especially suitable for dealing with nonlinear problems that are difficult to be solved by traditional search methods. In this paper, flow-focusing microchannel optimization was a complex mixed-variable nonlinear problem. Therefore, this paper used the NSGA-II to solve the multi-objective optimization problem. The flow chart of multi-objective optimization based on RBF and NSGA-II is shown in Figure 5.

Suppose $f_{1}\left(X\right)=D$,$\;f_{2}\left(X\right)=F$, and the objective function satisfies the following constraints:

(11) $$\{\begin{array}{c} Minimizef_{1}\left(x_{1},x_{2},x_{3},x_{4}\right)\\ Maximizef_{2}\left(x_{1},x_{2},x_{3},x_{4}\right) \end{array}$$

(12) $$\mathrm{subject}\;\mathrm{to}\{\begin{array}{c} \begin{array}{c} 0.5\leq x_{1}\leq 1.5\\ 1.5\leq x_{2}\leq 3.5 \end{array}\\ \begin{array}{c} 10\leq x_{3}\leq 30\\ 5\leq x_{4}\leq 15 \end{array} \end{array}$$

### 3.4. TOPSIS

In multi-objective optimization, selecting the final optimal solution from the Pareto frontier is important. The ideal point of the Pareto frontier is the optimal value for each objective, and the non-ideal point is the worst value for each objective. The technique for order preference by similarity to an ideal solution (TOPSIS) method can select the final optimal solution from the Pareto front [34].

The dimensions and scales of different objective spaces in multi-objective optimization problems should be unified, since the TOPSIS method used utilizes the Euclidean dimensionless objective $X_{ij}^{n}$, which is defined as

(13) $$X_{ij}^{n}=\frac{X_{ij}}{\sqrt[{2}]{\sum_{i=1}^{m}{\left(X_{ij}\right)}^{2}}}\left(i=1,2,\ldots ,m;j=1,2,\ldots ,n\right)$$

where $X_{ij}$ is the point on the Pareto front, m is the solution, and n is the target number.

In the TOPSIS method, the spatial distance between each solution on the Pareto boundary and the ideal point represented by $d_{i+}$ is determined as follows:

(14) $$d_{i+}=\sqrt[{2}]{\sum_{j=1}^{n}{\left(X_{ij}-X_{j}^{ideal}\right)}^{2}}$$

where $F_{j}^{ideal}$ is the ideal value of the jth objective obtained in the single-objective optimization.

The solution distance from the non-ideal point is denoted by $d_{i-}$. Therefore,

(15) $$d_{i-}=\sqrt[{2}]{\sum_{j=1}^{n}{\left(X_{ij}-X_{j}^{Non-ideal}\right)}^{2}}$$

where $F_{j}^{Non-ideal}$ is the non-ideal value of the jth objective obtained in the single-objective optimization.

The calculation of relative intimacy is shown as follows:

(16) $$r_{i}=\frac{d_{i-}}{d_{i+}+d_{i-}}$$

In the TOPSIS method, all solutions are ranked, and the final solution required is the solution with the maximum value; therefore, the compromise of the final selected solution is

(17) $$A_{best}=max\left(r_{i}\right)$$

## 4. Results and Discussion

### 4.1. Surrogate Model Accuracy Analysis

The RBF neural network training model uses 30 of the 40 sets of data from the sample space that the Latin hypercube sampled, while the post-training prediction model uses the remaining 10 sets. This post-training prediction model then necessitates an analysis of the microchannel design variables and the fitting accuracy of the objective function. The coefficient $R^{2}$ in Equation (18) was used in the paper to assess the fitting accuracy of the surrogate model. The maximum value of $R^{2}$ is 1, and the closer its value to 1, the better the fitting accuracy. The approximate model $R^{2}$≥ 0.9 was generally considered for engineering applications [35].

(18) $$R^{2}=1-\frac{\sum_{i=1}^{M}{\left(y_{i}-{\hat{y}}_{i}\right)}^{2}}{\sum_{i=1}^{M}{\left(y_{i}-{\bar{y}}_{i}\right)}^{2}}$$

where $y_{i}$ is the ith simulated value of the model, ${\hat{y}}_{i}$ is the ith predicted value of the model, ${\bar{y}}_{i}$ is the mean of the simulated values, and M is the number of samples.

**Figure 6:**
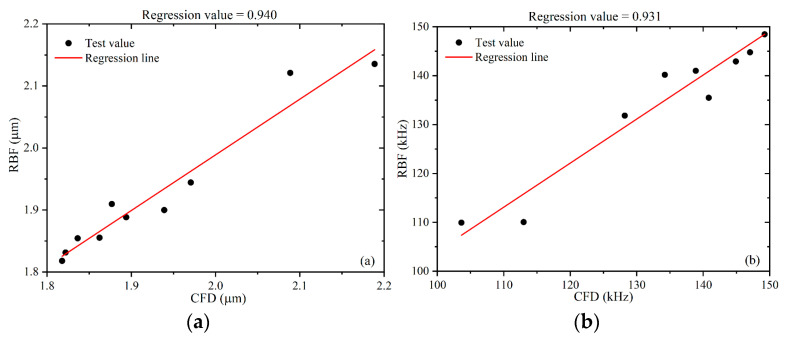
Comparison between prediction and reality: (**a**) D and (**b**) F.

It can be seen in Figure 6 the relationship between the simulated and predicted values of the flow-focusing microchannels by linearly fitting them; the evaluation coefficients $R^{2}$ are 0.940 and 0.931 for the two targets, respectively, based on the calculation of the above equations. The RBF predictions of the two objective functions are close enough to the computational fluid dynamics (CFD) test values, indicating that the developed RBF model is reliable and sufficient to design flow-focusing microchannel structures.

**Figure 7:**
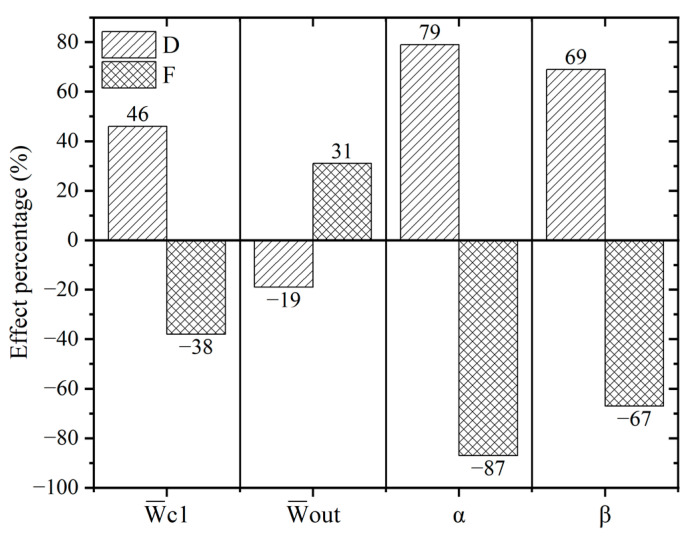
Sensitivity analysis.

In sensitivity analysis, the input variables of Pearson and Spearman linear correlation analysis are correlated with a certain objective function [36,37]. The results of the sensitivity analysis of the model are shown in Figure 7. As can be seen from Figure 7, the constriction angle is positively correlated with the microbubble diameter. It has the highest effect on the microbubble diameter (79%), followed by the expansion angle (69%) and the continuous phase inlet constriction width (46%). The outlet width has the least effect on the diameter (−19%), negatively correlated with the diameter.

For the generation frequency of microbubbles, the constriction angle was the most important influencing factor (−87%), negatively correlated with the generation frequency. This is followed by the expansion angle (−67%) and the continuous phase inlet constriction width (−38%). The outlet width has the least effect on the frequency (31%), positively correlated with the generation frequency.

### 4.2. Optimization Design Results

The optimization aims to obtain the best target values (highest generation frequency and minimum diameter). The diameter is measured by the microbubbles produced. The formation frequency is calculated from the time difference between the microbubbles’ separation from the orifice and the moment of the previous bubble separation. It can be seen from Figure 7 that all objective functions vary with different design parameters, and there exist design parameters corresponding to the optimal objective function. The Pareto optimal solution obtained by NSGA-II is necessary to represent when the problem to be addressed is targeted [38]. To detect the Pareto optimal frontier, the NSGA-II algorithm is used to calculate the optimal value. Table 2 shows the initial settings of the NSGA-II algorithm.

**Figure 8:**
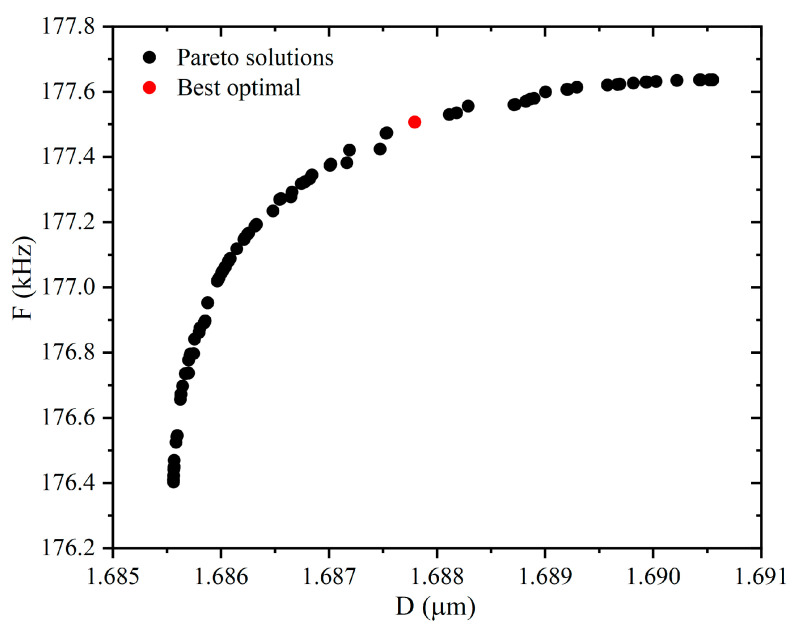
Pareto optimal front.

Figure 8 shows the Pareto optimal frontier diagram between the objective functions (red dots are optimal solutions). Each frontier solution represents the trade-off between the two objective functions. It is worth noting that the points on the Pareto front are optimal and are not dominated by each other. Each point corresponds to a specific structural parameter and optimization objective. To select the appropriate design point from many optimized design points to meet the requirements of practical engineering applications, TOPSIS is used to obtain the structural parameters corresponding to the final optimization results from the Pareto front, as shown in Figure 8. The objective values of the compromise solution are 1.6877 and 177.507, and the corresponding combinations of input parameters are ${\bar{W}}_{c1}$ = 0.9, ${\bar{W}}_{out}$ = 2.6, α = 10, and β = 5. The results of the error analysis of the predicted results with the numerical simulation show that the relative errors of the diameter and frequency are 0.43% and 1.17%, respectively, as shown in Table 3. This indicates that the predicted values are consistent with the CFD calculation results. The results show that the multi-objective optimization method based on RBF, NSGA-II, and TOPSIS proposed in this paper is effective.

### 4.3. Performance Evaluation of Flow-Focusing Microchannels

Most research efforts have focused on fluid properties and operating conditions for traditional flow-focusing microchannels. In this paper, improved performance was achieved by enhancing the flow-focusing microchannel, and the performance of the improved microchannel is compared with that of the traditional microchannel [22]. To be consistent with the structural parameters of this paper, the orifice width of the traditional microchannel is 2 μm, and the continuous phase inlet width is set to 4 μm.

#### 4.3.1. Flow Regimes in Flow-Focusing Microchannels

**Figure 9:**
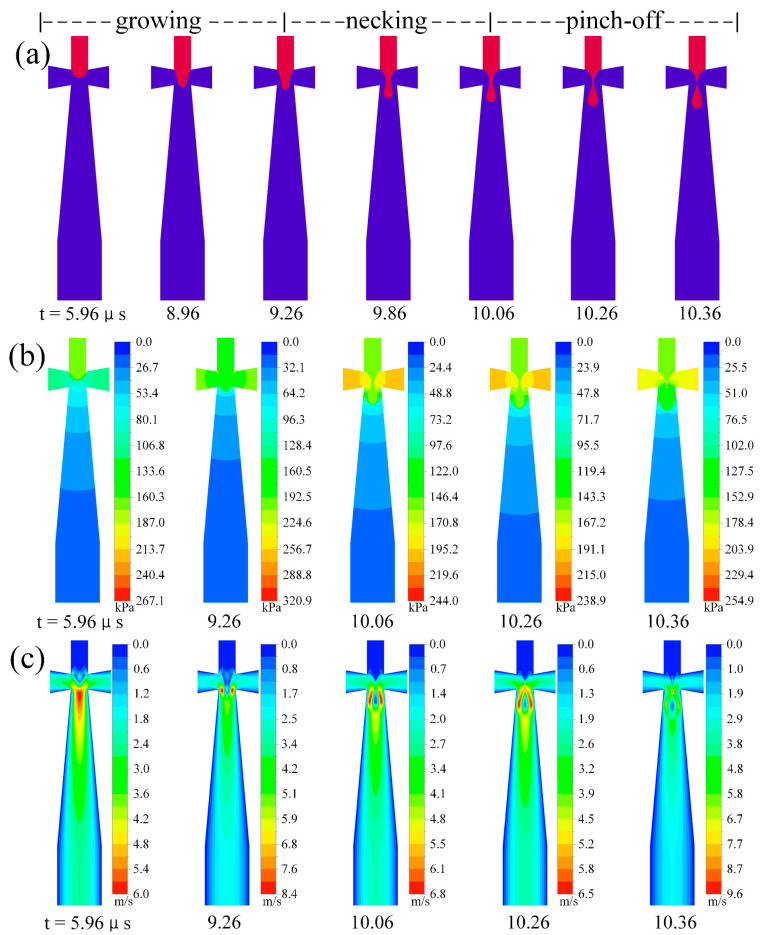
Time evolution of hydrodynamic information during microbubble generation in improved microchannel (Ca = 0.0139, U_d_ = 0.2 m/s): (**a**) evolution of the interface, (**b**) evolution of pressure field, and (**c**) evolution of velocity field.

**Figure 10:**
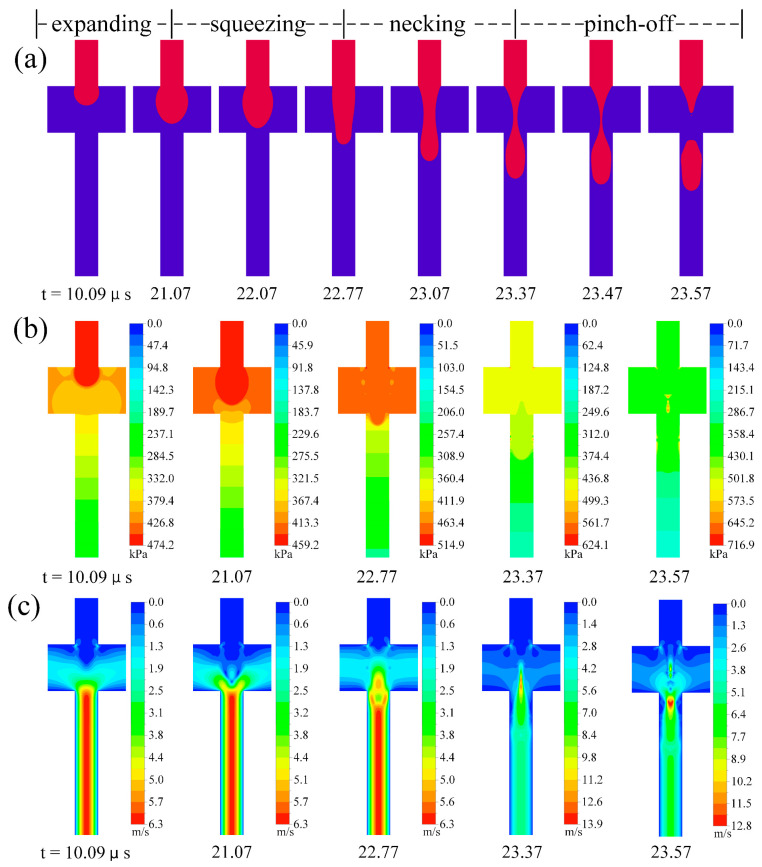
Time evolution of hydrodynamic information during microbubble generation in traditional microchannel (Ca = 0.0139, U_d_ = 0.2 m/s): (**a**) evolution of the interface, (**b**) evolution of pressure field, and (**c**) evolution of velocity field.

Figure 9 and Figure 10 show the time evolution of the fluid dynamics information during microbubble generation in two flow-focusing microchannels. The figures include two flow states: dripping and squeezing regime, showing different fluid flow properties and microbubble generation performance. Microbubble generation mechanisms are essential for understanding both microchannels; therefore, these effects are discussed in both microchannels using flow field profiles.

Figure 9 shows the time evolution of the hydrodynamic information during microbubble generation in the improved microchannel, where the flow regime is in the dripping regime. The process of microbubble generation in the dripping regime can be divided into three stages: growing, necking, and pinch-off. The initial phase of the first microbubble generation can be seen in the dripping regime (see t = 5.96–9.26 μs in Figure 9), where the front part of the dispersed phase threads mainly stretches toward the main downstream channel without significant expansion in the direction of the side channels. As the thread continues to extend into the main downstream channel, the transverse interface of the thread becomes concave. The necking phase (see t = 9.26–10.06 μs in Figure 9) begins with the formation of a neck, which contributes to necking and thread extension due to the enhanced squeezing of the continuous phase under the action of the constriction inlet, in combination with the viscous shearing effect of the continuous phase. After the necking stage, a pinch-off stage occurs (see t = 10.06–10.36 μs in Figure 9), where high pressure appears on both sides of the neck, and the pressure difference is generated with the neck further promoting the necking. It can be seen that the fragmentation and generation of microbubbles occur at the junction of two phases. Due to the improved continuous phase inlet channel in this paper, it will be found that the microbubble diameter is smaller than the channel width. In this flow state, microbubbles’ generation is consistent, stable, and uniform.

Figure 10 shows the time evolution of the hydrodynamic information during microbubble generation in a traditional microchannel where the flow regimes are in the squeezing regime. The process of microbubble generation in the squeezing regime can be divided into four stages: expanding, squeezing, necking, and pinch-off. During the expanding stage (see t = 10.09–21.07 μs in Figure 10), the front part of the dispersed phase continuously swells with the continuous injection of the dispersed phase and remains spherical at the intersection. As the front area increases, the gap between the interface and corners of the main and side channels decreases, thus limiting the continuous phase flow into the main downstream channel. A squeeze will form in the dispersed phase, and the squeezing phase begins. During the squeezing stage (see t = 21.07–22.77 μs in Figure 10), the thread tip starts to thin and gradually moves downward under continuous phase squeezing, blocking the downstream exit of the intersection. The convex surface of the front gradually flattens out, followed by the necking stage. At this stage (see t = 22.77–23.37 μs in Figure 10), the thread plane starts to change to a concave surface, and a neck is formed at the intersection. Under the squeezing pressure of the continuous phase, the neck continuously shrinks while the thread stretches downstream and then enters the pinch-off phase (see t = 23.37–23.57 μs in Figure 10). As the continuous phase constantly squeezes the neck, the neck rapidly contracts, and pinch-off occurs. The fragmentation and generation of microbubbles occur near the intersection of the aqueous and gas phases. The generated microbubbles become clogged in the outlet channel and encounter higher resistance to move downstream.

The time-dependent pressure and velocity contours near the intersection allow us to understand the generation of microbubbles under different microchannels. It is clear from the pressure contours (Figure 9 and Figure 10) that the pressure in the main channel is decreasing in sequence. According to the Laplace pressure theory, the surface tension between the two phases causes the pressure to jump at the curved interface, and the internal pressure of the microbubble is higher than the internal pressure of the surrounding stable phase. It can be seen that the gas continuously enters the main channel; as the water phase constantly squeezes the gas, there is a pressure drop; this pressure drop plays an essential role in the fragmentation of microbubbles. In the microbubble pinch-off phase, a high-pressure region can be observed near the separation point (see t = 23.57 μs in Figure 10b).

For the velocity contours, the maximum velocity in the improved and traditional microchannels during fragmentation occurs during the pinch-off stage (see t = 10.36 μs in Figure 9c and t = 23.37 μs in Figure 10c). Comparing the two microchannels, it can be seen that the maximum velocity reached in the traditional microchannel is 13.9 m/s. The velocity at the orifice is higher in the improved microchannel, and the outlet channel is higher in the traditional microchannel.

**Figure 11:**
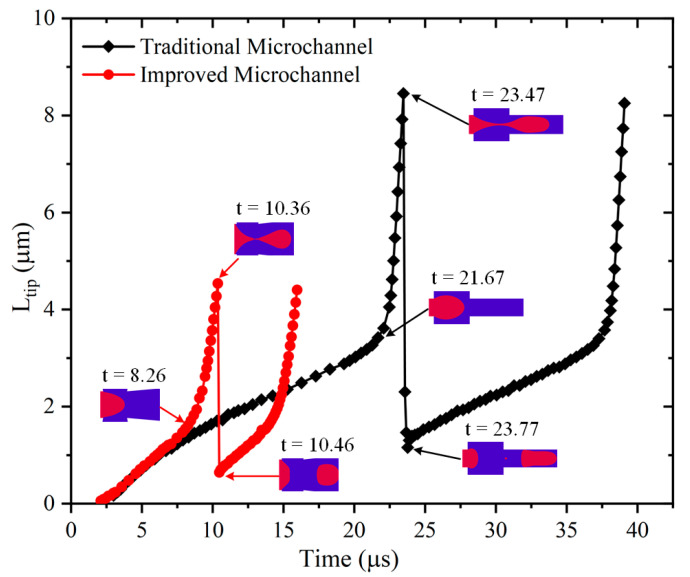
Variation of tip length with time during formation of microbubbles in two microchannels.

The evolution of the thread profile helps understand the fluid dynamics behind the generation of microbubbles in different microchannels. Figure 11 shows the variation of tip length over time during the formation of microbubbles in both microchannels; $L_{tip}$ is the distance from the threaded tip to the end of the main inlet channel in the connection region (see Figure 1).

For the improved microchannel, the $L_{tip}$ tip increases as the dispersed phase threads stretch downstream during the growth phase in the dripping regime. At t = 8.26 μs, it can be seen that the enhancement of the continuous phase squeeze leads to an acceleration of the downstream motion of the thread tip. After the first microbubble is generated, a significant retraction of the remaining thread tip can be seen. After 10.46 μs, the tip of the dispersed phase gradually increases and enters the next cycle of microbubble generation. For the traditional microchannel, the change in the $L_{tip}$ tip is similar to that of the improved microchannel, and the slope of the change in the $L_{tip}$ tip is the same in both microchannels before the inflection point acceleration occurs. After 21.67 μs, the tip of the thread rapidly stretches downstream, and after the neck pinch-off, it can be seen that the tip of the remaining thread and the tail of the newly formed microbubble quickly retract due to the interfacial tension (t = 23.47–23.77 μs), after which the next cycle of microbubble generation begins. Compared with the traditional microchannel, the maximum $L_{tip}$ and minimum retraction $L_{tip}$ of the improved microchannel are both smaller.

#### 4.3.2. Pressure Changes during Microbubble Formation

**Figure 12:**
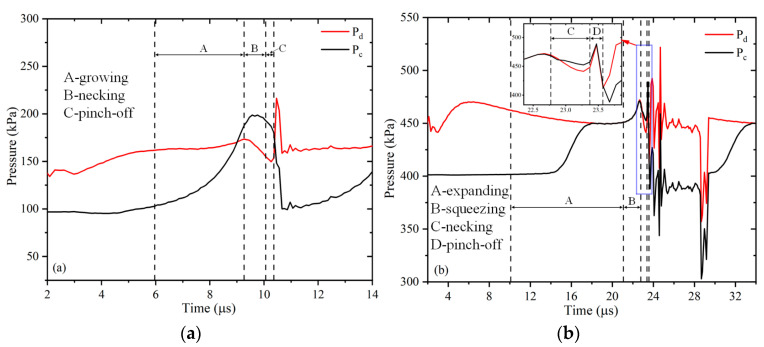
Pressure curve: (**a**) improved microchannel; (**b**) traditional microchannel (Ca = 0.0139, U_d_ = 0.2 m/s).

The contribution of pressure in different microchannels to the fragmentation mechanism during microbubble generation can be further investigated by observing the pressure gradient with time near the orifice [39]. The pressure variation near the orifice helps to understand the fundamental physical process of microbubble generation. Figure 12 shows the variation of the two-phase (dispersed phase and continuous phase) pressure with time for the two microchannels. $P_{c}$ is the pressure at the continuous phase channel connection point c, and $P_{d}$ is measured at point d before the main channel connection point.

In both microchannels, pressure changes exhibit similar behavior on the time scale. The improved microchannel (Figure 12a) has $P_{d}$>$P_{c}$ at the very beginning, and as the gas enters, it will appear that $P_{c}$ > $P_{d}$. Initially, the gas continuously enters the main channel, and $P_{c}$ remains approximately stable. As the gas continues to enter, the pressure accumulates, $P_{c}$ rises, and the pressure rise slows down at 9.26 μs when the microbubble neck begins to contract. At t = 10.36 μs, the gas ruptured to form microbubbles, followed by a rapid decrease in $P_{c}$. For $P_{d}$, first, the gas keeps entering the main channel, and $P_{d}$ keeps increasing. After the gas enters the crossover, it is subjected to resistance from the liquid phase, and the pressure basically remains constant. Before 9.26 μs, $P_{d}$ starts to be smaller than $P_{c}$, and there is a necking trend. At 9.26 μs, the neck starts to constrict, and $P_{d}$ starts to decrease. $P_{d}$ rapidly increased after microbubble fragmentation, and then $P_{c}$ and $P_{d}$ started to fluctuate. In the traditional microchannel (Figure 12b), it can be seen that, initially, $P_{c}$>$P_{d}$, and as the gas continues to enter, $P_{d}$ first increases and then slowly decreases. $P_{c}$ remains stable at first and then starts to increase until it is in line with $P_{d}$. In the squeezing stage, $P_{d}$ = $P_{c}$. After t = 22.77 μs, $P_{c}$ starts to be larger than $P_{d}$ after the start of necking. At t = 23.57 μs, the gas ruptures to form microbubbles, and $P_{d}$ rapidly rises and $P_{d}$ > $P_{c}$. After that, $P_{d}$ and $P_{c}$ fluctuate. It is clear that the average pressure value in the improved microchannel is lower than in the traditional one.

**Figure 13:**
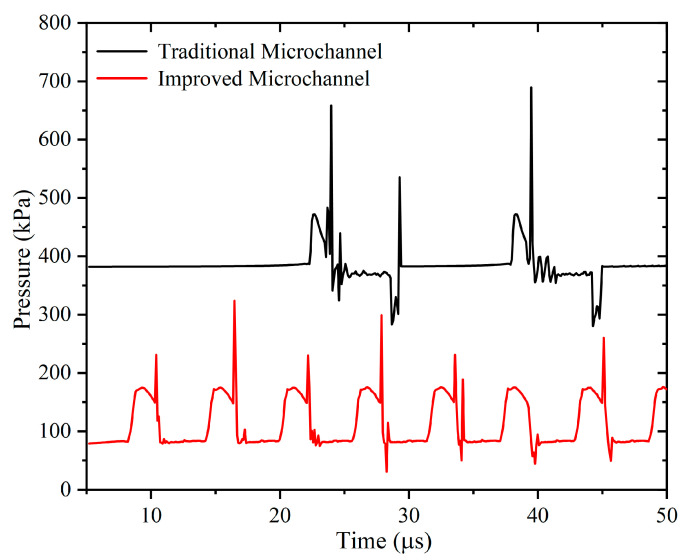
Variation of pressure at p-point with time in traditional and improved microchannels.

The pressure fluctuations at the inlet of the main channel shown at the p-point during microbubble generation in traditional and improved microchannels are given in Figure 13. The curve indicates that the pressure remains constant at the beginning and then periodically fluctuates. Usually, during the formation of each microbubble, the pressure increases and then decreases, so it can be seen from the graph that there are many peaks in the pressure fluctuations, and each peak represents the production of one microbubble. The pressure at the p-point in the improved microchannel is lower than that in the traditional microchannel, and the pressure more rapidly changes in the improved microchannel.

#### 4.3.3. Microbubble Diameter and Generation Frequency

**Figure 14:**
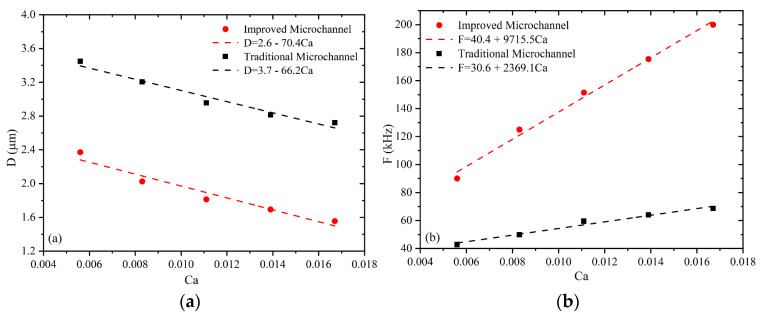
(**a**) Microbubble diameter (D) and (**b**) generation frequency (F) as a function of capillary number (Ca) (U_d_ = 0.2 m/s).

Figure 14a shows the variation of the microbubble diameter for different capillary numbers Ca, where D denotes the area-equivalent circle diameter. The figure shows that the microbubble diameter decreases with increasing Ca number. This reduction is due to the injection of the continuous phase at a higher rate, resulting in smaller microbubbles. It is clear from the figure that the microbubbles under the traditional flow-focusing microchannel are larger than the channel width, whereas the microbubbles under the improved flow-focusing microchannel are smaller than the channel width. The smallest microbubbles were from the improved flow-focusing microchannel at Ca = 0.0167, whereas the largest microbubbles were observed in the blocked state under the traditional flow-focusing microchannel at Ca = 0.0056. By fitting the microbubble diameter with the number of capillaries, the slope of the linear fitting function of the traditional microchannel is smaller than that of the improved microchannel. It can be seen that the diameter of the microbubble generated by the improved microchannel will be smaller with the increase in the number of capillaries.

Figure 14b shows the frequency of microbubble generation for different capillary numbers. The generation frequency is inversely proportional to the microbubble fragmentation time, which is the moment when the gas phase is separated from the two lateral channels by the water phase. When the water flow is injected into the connection at a higher velocity, the higher the Ca number, the shorter the microbubble fragmentation time, and vice versa, the higher the microbubble generation frequency. The microbubble generation frequency was fitted to the capillary number. Moreover, the slope of the increase in generation frequency with increasing Ca number in the improved microchannel was much larger than that in the traditional microchannel, as seen from the fitted linear function. At the same Ca number, the generation frequency of the improved microchannel is greater than that of the traditional microchannel. At Ca = 0.0167, the maximum frequency is 200 kHz in the improved microchannel and 68.617 kHz in the traditional microchannel. At Ca = 0.0056, the minimum frequency in the improved microchannel is 90.09 kHz, and the minimum frequency in the traditional microchannel is 42.765 kHz. As shown in Table 3, the constriction of the continuous phase inlet enhanced the flow-focusing effect; the microbubble diameter of the improved microchannel decreased from 2.8141 μm to 1.6949 μm, and the microbubble generation frequency increased from 64.077 kHz to 175.438 kHz compared with the traditional microchannel when Ca = 0.0138.

## 5. Conclusions

In this paper, numerical simulations and optimization algorithms investigated the fluid flow characteristics of microbubble generation in an improved flow-focusing microchannel with a constricted continuous phase inlet. A two-dimensional numerical model of microbubble generation in a flow-focusing microchannel was developed using the VOF method. Four design variables were obtained by the Latin hypercube sampling method, and the RBF surrogate model was used to establish the relationship between the objective function and design variables. The $R^{2}$ of the microbubble diameter and generation frequency were 0.940 and 0.931, respectively, which indicated that the RBF surrogate model could respond well to the relationship between the design variables and corresponding objective function. The results of the sensitivity analysis showed that the contraction angle had the most significant effect on the diameter with a positive correlation, and the outlet width had the least impact on the diameter with a negative correlation. The contraction angle has the most significant effect on the generation frequency with a negative correlation, followed by the expansion angle, continuous phase inlet contraction width, and outlet width, where the outlet width is positively correlated with the generation frequency. Then the optimal design of the NSGA-II algorithm was carried out, and TOPSIS decision analysis was performed on the obtained Pareto solution set to obtain the final optimal result of the microbubble diameter of 1.6949 μm and generation frequency of 175.438 kHz. Finally, the fluid flow characteristics during microbubble generation in the improved and traditional flow-focusing microchannels were investigated. It was found that the flow regime of microbubbles generated by the improved microchannel is dripping, whereas the flow regime of microbubbles caused by the traditional microchannel is squeezing. The pressure at the orifice in the improved flow-focusing microchannel reaches its maximum earlier in the whole microbubble formation cycle, which contributes to the separation of microbubbles more than the traditional microchannel. Constriction of the continuous phase inlet enhances the flow-focusing effect. When the Ca numbers are the same, the generated microbubbles in the improved flow-focusing microchannels have smaller diameters and higher frequencies; the microbubble diameters in both microchannels decrease with increasing Ca numbers, and the generation frequencies increase with increasing Ca numbers. According to the fitted linear function, it is known that the slope of the decrease in the microbubble diameter with increasing Ca number and the slope of the increase in microbubble generation frequency with increasing Ca number are greater in the improved microchannel compared with the traditional one. We hope that the results of the study of flow-focusing microchannels will provide guidance for the design of microbubble generators for high frequencies. In this paper, the reliability of the microchannel simulations was confirmed by correct grid independence checks and model validation, and the analytical procedure and numerical results used help to provide guidance for the design of microbubble generators at high frequencies. The necessary experiments are important for the study of the microbubble generation characteristics, and we will make experiments a focus of future research.

## Figures and Tables

**Table 1:** Design variables and design space.

Limit	${\bar{W}}_{c1}$	${\bar{W}}_{out}$	α	β
Upper	1.5	3.5	30	15
Lower	0.5	1.5	10	5

**Table 2:** Parameters setting of NSGA-II.

Parameters	Values
Population size	12
Number of generations	100
Crossover probability	0.9
Crossover distribution index	10.0
Mutation distribution index	20.0

**Table 3:** Comparison between numerical and RBF predicted values.

Design	Diameter (μm)	Frequency (kHz)
Improved microchannel	1.6949	175.438
Traditional microchannel	2.8141	64.077
Relative error (%)		
Im (simulation and predictive)	0.43	1.17

## Appendix A

**Table A1:** Latin hypercube sampling method for generating 40 data samples within CFD.

No.	Design Variables				Objective Functions	
	${\bar{W}}_{c1}$	${\bar{W}}_{out}$	α	β	D	F
1	1.2	2.2	11	9	1.8939	138.888
2	1.4	1.5	26	12	2.1013	112.994
3	0.9	2.6	23	12	1.7746	156.250
4	1.1	2.5	13	8	1.8194	149.253
5	0.9	2.7	10	6	1.7054	173.913
6	0.8	3.4	30	14	1.9273	134.228
7	1.4	2.8	24	10	2.0886	112.994
8	0.9	3.5	18	13	1.8610	143.884
9	1.0	3.0	20	14	1.8219	149.253
10	0.8	2.3	17	13	1.9165	136.986
11	1.2	2.0	16	10	1.9321	136.986
12	1.3	2.1	24	12	2.0200	124.223
13	1.4	3.3	23	8	2.0623	114.942
14	0.8	3.3	25	9	1.8178	149.253
15	1.0	2.5	14	14	1.7962	151.515
16	1.4	1.7	17	5	2.0995	116.279
17	0.6	2.2	28	5	1.8103	152.671
18	0.5	1.6	21	7	1.8769	140.845
19	1.2	2.4	29	15	1.9197	133.333
20	1.5	3.1	14	6	2.1888	103.626
21	1.1	2.7	22	9	1.8060	151.515
22	1.0	2.0	25	11	1.7564	158.730
23	1.2	1.8	15	12	1.9391	134.228
24	0.8	1.8	28	10	1.8421	147.058
25	1.5	3.2	27	15	2.2303	101.010
26	0.6	3.0	22	7	1.8623	144.927
27	0.7	2.6	19	8	1.8363	147.058
28	0.7	2.4	19	8	1.8535	144.927
29	1.3	2.9	21	11	2.0171	124.223
30	1.3	1.6	12	7	1.9769	128.205
31	0.7	3.2	15	10	1.9033	138.888
32	0.5	1.9	12	7	1.8604	140.845
33	1.3	2.9	20	6	1.9706	128.205
34	0.6	2.8	13	6	1.8337	147.058
35	0.9	3.4	18	13	1.8764	143.884
36	0.7	1.9	29	13	1.9444	131.578
37	1.1	2.1	26	11	1.8311	149.253
38	1.0	1.7	16	14	1.8022	150.375
39	0.6	3.1	27	11	1.9577	130.718
40	1.1	2.3	11	9	1.8286	149.253
